# Enzymatic Pretreatment of Plant Cells for Oil Extraction

**DOI:** 10.17113/ftb.61.02.23.7896

**Published:** 2023-06

**Authors:** Hanna Vovk, Kwankao Karnpakdee, Roland Ludwig, Tamara Nosenko

**Affiliations:** 1 Educational and Scientific Institute of Food Technology, Department of Fats, Perfumery and Cosmetic Products Technology, National University of Food Technologies, Kyiv, Volodymyrska street 68, 01601 Kyiv, Ukraine; 2 Institute of Food Technology, Department of Food Science and Technology, University of Natural Resources and Life Sciences (BOKU), Vienna, Muthgasse 18, 1190 Vienna, Austria

**Keywords:** pretreatment, hydrolases, oil extraction, oilseeds, pressing

## Abstract

Oil from oilseeds can be extracted by mechanical extraction (pressing), aqueous extraction, or by extraction with organic solvents. Although solvent extraction is the most efficient method, organic solvents are a potential hazard to the life and health for workers as well as to the environment, when solvent vapours are released and act as air pollutant with a high ozone-forming potential. Pressing is safer, environmentally friendly, and it preserves valuable natural components in the resulting oils. The problems associated with pressing are the high energy consumption and the lower yield of oil extraction, because the applied mechanical force does not completely destroy the structural cell components storing the oil. In seed cells, the oil is contained in the form of lipid bodies (oleosomes) that are surrounded by a phospholipid monolayer with a protein layer on the surface. These lipid bodies are further protected by the seed cell walls consisting mainly of polysaccharides such as pectins, hemicelluloses and cellulose, but also of glycoproteins. The use of hydrolases to degrade these barriers is a promising pretreatment strategy to support mechanical extraction and improve the oil yield. It is advisable to use a combination of enzymes with different activities when considering the multicompartment and multicomponent structure of oilseed cells. This article gives an overview of the microstructure and composition of oilseed cells, reviews enzymes capable of destroying oil containing cell compartments, and summarizes the main parameters of enzymatic treatment procedures, such as the composition of the enzyme cocktail, the amount of enzyme and water used, temperature, pH, and the duration of the treatment. Finally, it analyzes the efficiency of proteolytic, cellulolytic and pectolytic enzyme pretreatment to increase the yield of mechanically extracted oil from various types of vegetable raw materials with the main focus on oilseeds.

## INTRODUCTION

With the development of technology, the food market now mostly supplies processed foods to serve the consumer’s convenience. Processing, however, cannot only be used to prepare food for the consumer benefit, but also to save costs on raw materials and to replace expensive natural components by cheaper synthetic ones. Sometimes valuable components are also degraded or lost by harsh processing methods or conditions. Vitamins, minerals, amino acids and polyunsaturated fatty acids are key components of a balanced diet that prevent the deterioration of health and decrease the incidence of obesity, diabetes, cardiovascular disease and cancer. Vegetable oils are an especially valuable source of biologically active substances with useful properties in the treatment and prevention of many diseases (*1*-*4*). These substances include natural antioxidants, ω-6 and ω-3 fatty acids, which are represented by linoleic and linolenic fatty acids, respectively, as well as phytosterols and squalene – a precursor to the formation of sterols, steroid hormones and vitamin D (*1*-*6*).

The content of these substances in oil depends on the raw materials and the oil extraction process. The two most common techniques to extract oil from oilseeds are mechanical extraction by using a press (pressing) and oil extraction by using an organic solvent. Although solvent extraction is more efficient and allows to extract more oil from the raw material than pressing, this technology involves the use of organic solvents (mostly hexane), which is dangerous in the production process (flammable and explosive) and a risk for human health if not properly removed from the product (*3*, *7*, *8*). In addition, the release of solvent vapours into the environment is also hazardous from an ecological point of view, as they react with air pollutants and form ozone and photochemicals (*7*). Finally, the oil obtained by the extraction method must be refined, which causes the loss of many biologically active substances.

The pressing method is more environmentally friendly (despite its high energy consumption) and safe and it also helps to preserve valuable natural components in the resulting oils (especially cold pressing). However, pressing does not allow to extract oil from the oilseeds completely and a significant percentage remains in the cake. This is the reason of the ongoing research to intensify mechanical oil extraction technologies (*3*). Some intensification approaches include solvent extraction methods that use terpenes and ionic liquids as green solvents, but the most promising among these methods is the enzymatic pretreatment of plant materials and especially oilseeds with hydrolases before pressing (*3*, *4*, *7*, *9*-*11*).

The oil within oilseeds is very well protected by compartimentalization and various structural components within the oilseed cells. In order to release the oil from its subcellular and cellular compartments, it is necessary to degrade these structural components. To support mechanical force that by itself does not result in a complete extraction, the use of hydrolytic enzymes, which are fast and specific catalysts, is promising to achieve a high oil yield by partial hydrolysis of various oil material cell constituents.

## MICROSTRUCTURE AND COMPOSITION OF OILSEED CELLS

The lipid bodies, which contain the oil in the seed cells, are called oleosomes (*8*, *12*-*16*). Oleosomes are droplets formed by triacylglycerides and surrounded by a membrane consisting of a monolayer of phospholipids and a surface protein layer that penetrates the phospholipid layer and reaches into the oleosome (*15*, *16*). The phospholipid layer is formed mainly by phosphatidylcholine and phosphatidylserine, while oleosin predominates in the protein layer together within lower amounts of kaleosin and steroleosin (*14*, *15*).

The structure of the plant cell wall is formed by linear cellulose chains and branched hemicellulose chains immersed in a lignin matrix and features cross-linking lignin–carbohydrate bridges, ether, and C–C bonds (*17*). The cell walls of oilseeds consist mainly of polysaccharides such as cellulose, hemicelluloses, lignin, pectins and arabinogalactans, but also contain a low amount of hydroxyproline-rich glycoproteins (*12*, *18*). Cellulose is a linear chain of β-1,4-linked d-glucose units, which can be cleaved by various cellulases (Table 1 (*17*-*41*)). Hemicelluloses are linear or branched homo- or heteropolysaccharides bound to cellulose microfibrils by hydrogen bonds or connected to lignin by covalent bonds and thereby form the complex and solid structure of plant cell walls.

**Table 1:** Microstructure, carbohydrate composition and enzymes proposed to be degrading oleaginous material cell walls

Oilseed or oil-containing material cell wall microstructure	Image description	Cell wall constituting oligo- and polysaccharides	Corresponding enzymes for oligo- and polysaccharide degradation	Ref.
Soybean seed 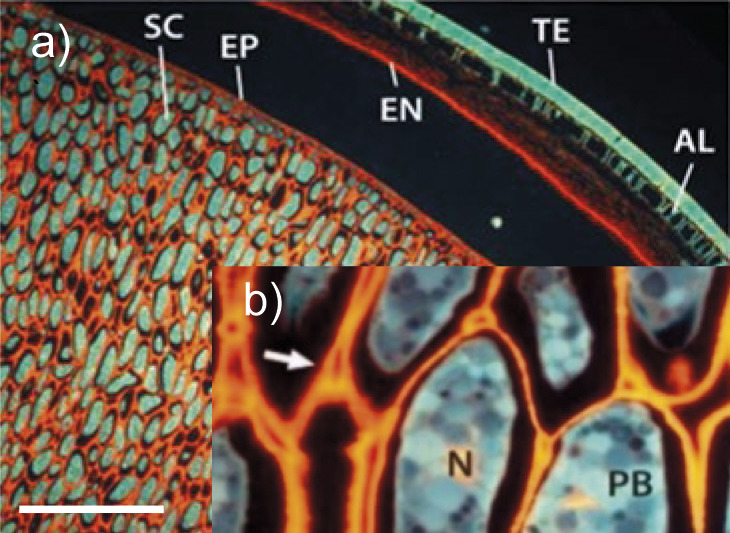	General seed microstructure of intact soybean tissue. SC=storage cotyledons, TE=testa (seed coat), EP=epidermis, EN=endosperm, AL=aleurone layer, PB=protein bodies, N=nucleus, arrow=pectin-rich region. *l*(bar)=500 µm (image a). A close up-of the cell wall is shown in the inset (image b). The copyright for this picture is granted by CC BY 4.0.	Cellulose, mannan, galactan, arabinan, xyloglucan, rhamnogalactu-ronans, arabino-galactan I, xylo-galacturonan, galacturonan, homogalacturonan	Enzymes for cellulose and hemicellulose destruction, xyloglucan-degrading enzymes, arabinogalactan-degrading enzymes, pectolytic enzymes, rhamnogalacturonan-degrading enzymes	(*17*-*20*, *24*-*35*)
Rapeseed 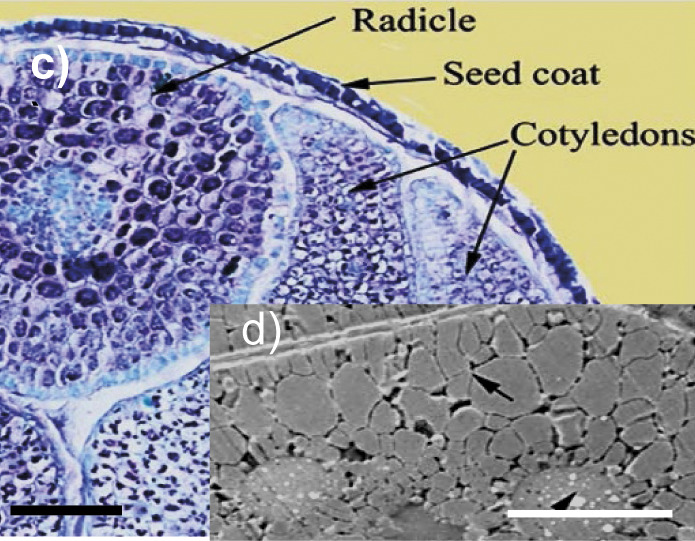	Seed coat, radicle and cotyledons of a mature rapeseed of ultrahigh oil content *Brassica napus* line YN171 (image c). Ultrastructure of the rapeseed cotyledon cell with the protein bodies (arrowhead) and oilbodies (arrow) (image d). *l*(bar)=200 µm (image c), *l*(bar)=5 µm (image d). The copyright is granted by CC BY 4.0.	Cellulose, hemicelluloses, arabinan, arabinogalactan, pectins	Enzymes for cellulose and hemicellulose destruction, arabinogalactan-degrading enzymes, pectolytic enzymes, rhamnogalacturonan-degrading enzymes	(*17*-*20*, *26*-*32*, *36*, *37*)
Hemp seed 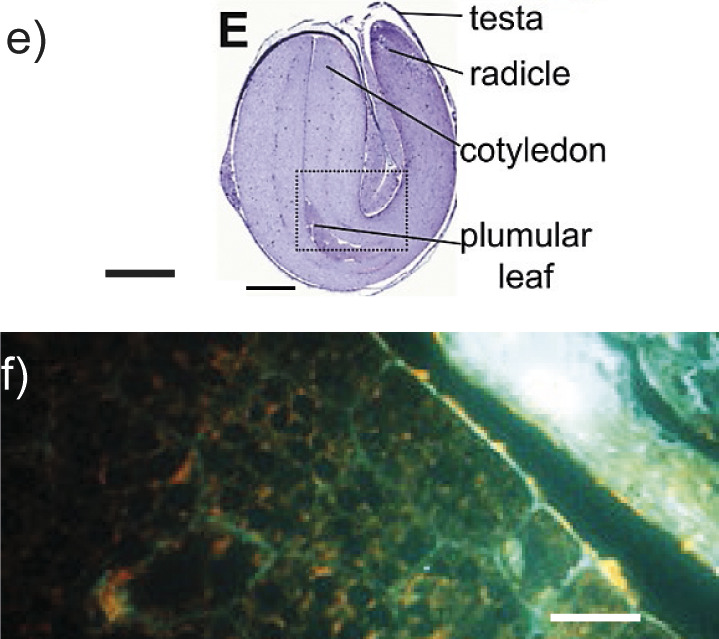	Transverse section of hemp heart stained with toluidine blue (image is an *in silico* “stitch” of 15×15 images without other modifications). Hemp heart tissue stained to reveal cell walls (cellulose/calcofluor white) (image e). Hemp cotyledon stained with calcofluor white and showing autofluorescence (image f). *l*(bar)=1 mm (image e) and 20 μm (image f). The reproduction copyright is granted by Elsevier.	Unesterified and low esterified homogalacturonan, rhamnogalacturonan I, arabinogalactan proteins, callose, polysaccharides containing α-1,5-l-arabinan	Enzymes for hemicellulose degradation, arabinogalactan-degrading enzymes, pectolytic enzymes, rhamnogalacturonan-degrading enzymes	(*17*-*20*, *22*, *26*, *28*-*32*)
Rice bran 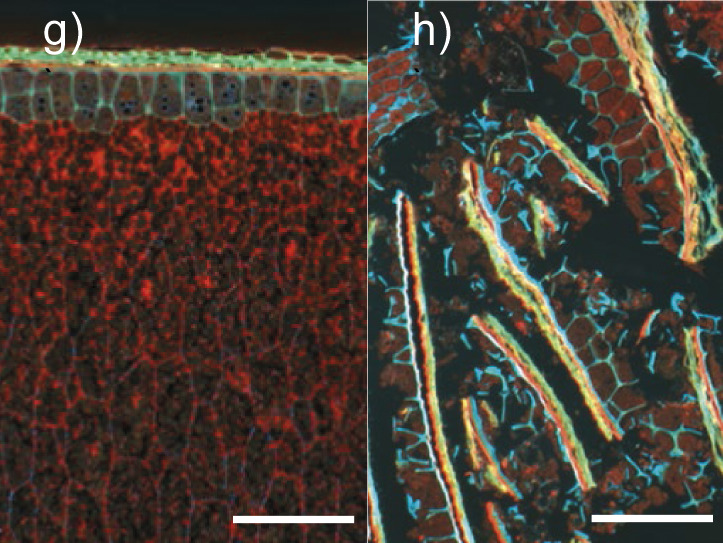	Microstructure of rice grain outer layers (image g). Fresh, non-defatted, and non-milled rice bran. Cell wall glucans stained with calcofluor appear blue and proteins stained with acid fuchsin appear red, pericarp structures appear yellowish due to autofluorescence, starch is unstained and appears black (image h). *l*(bar)=100 µm (images g and h). The copyright is granted by CC BY 4.0.	Cellulose, hemicelluloses, arabinoxylans, β-glucan, pectins	Enzymes for cellulose and hemicellulose destruction, arabinogalactan-degrading enzymes, pectolytic enzymes, rhamnogalacturonan-degrading enzymes	(*17-21,23,* *26-32, 38,39)*
*Moringa oleifera* seed 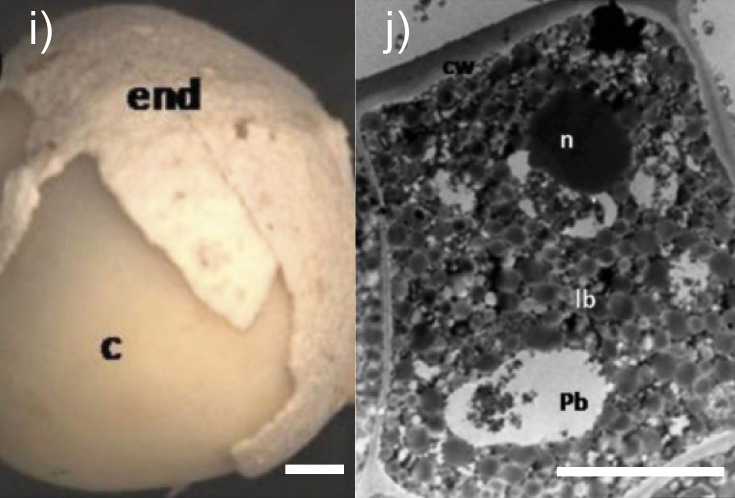	*Moringa* seed morphology. Seed with partially removed internal seed coat. Cotyledon (c), endotesta (end) (image i). Sections of a fresh *Moringa oleifera* seed (cotyledon). Epidermal cell of the cotyledon with lipid bodies (lb), fewer protein bodies (pb) and a thick external cell wall (image j). *l*(bar)=1 mm (image i) and 5 µm (image j). The copyright is granted by CC BY 4.0.	Cellulose, arabinogalactan, xylan-type polysaccharides	Enzymes for cellulose and hemicellulose destruction, xyloglucan-degrading enzymes, arabinogalactan-degrading enzymes	(*17*, *18*, *20*, *26*-*29*, *32*-*35*, *40*, *41*)

Hemicelluloses consist of a large number of different mono- and oligosaccharides, including glucans, xylans, mannans, galactans, xyloglucans, arabinogalactans, *etc.* Based on their composition, arabinogalactans belong to the hemicelluloses. Despite not containing galacturonic acid residues, some authors also refer to arabinogalactans as pectins, due to their presence in the hair regions of pectin chains in the form of neutral sugars (*19*, *20*, *42*). Some arabinogalactans form compounds with proteins called arabinogalactan proteins (*20*). Due to the substrate specificity and regiospecificity of each hemicellulase, the destruction of a particular hemicellulose (Table 1) is best performed by a mixture of enzymes (*18*).

Lignin is a phenolic macromolecule consisting of *p*-hydroxyphenyl (H), guaiacyl (G) and syringyl (S)-type methoxylated phenylpropane units connected by different types of carbon–carbon and ether linkages, which is found in the cell walls of vascular plants (*21*, *43*). Several enzymes are known to attack and degrade lignin, such as laccase (EC 1.10.3.2), lignin peroxidase (LiP, EC 1.11.1.14), manganese peroxidase (MnP, EC 1.11.1.13), and versatile peroxidase (VP, EC 1.11.1.16)). Laccase and peroxidases catalyze depolymerization of the lignin macromolecules with formation of phenolic hydroxyl groups, oxidation of phenolic groups to phenoxy radicals and subsequent cross-linking of lignin (*17*, *43*).

## ENZYMATIC DEGRADATION OF CELL WALL COMPONENTS

In nature, phenol-oxidizing enzymes are produced by basidiomycetes such as white-rot fungi, but also brown-rot fungi. White-rot fungal enzymes are capable of destroying not only lignin, but also all the main components of lignocellulose, including cellulose and hemicellulose (*17*, *21*, *43*, *44*). The enzymes expressed by brown-rot fungi are able to cause lignin oxidation, depolymerization, demethylation of lignin methoxy groups, and the removal of cellulose and hemicelluloses from plant cell walls (*17*, *43*).

The technology of enzyme-assisted delignification can be used to process lignocellulosic materials into medium-density fibre board and particle board, aromatic value-added chemicals, biofuels, and in paper pulp manufacture (*17*, *21*, *43*). However, is it appropriate to use phenol-oxidizing enzymes to destroy the cell walls of oilseeds to increase oil yield? Lignin is a main component of the seed coat. The content of lignin and cellulose carbohydrates in the seed kernels is much lower than in the husks. The compositional analysis of 20 hemp seed varieties and lines revealed that the presence of these compounds in the hemp seed hulls ranged from mass fraction of 16.0 to 19.5 % for lignin and from 22.0 to 36.7 % for cellulose, while both of these components were absent from the hemp seed kernel (*22*). The lignin mass fraction in rice bran was in the range of 7.7–24.8 %, in the seed coats of rapeseed, sunflower and cucurbit seeds was from 4.8 to 27.1 %, while the mass fraction of lignin in the oilseed kernels varied from 0.5–0.6 % for rapeseed and sunflower seeds and to 2.0–2.9 % for cucurbit seeds (*23*, *45*-*57*). It is important to consider that enzymes catalyzing the oxidation of lignin can also modify the oil, which is highly undesirable. It was found that MnP and VP are able to cause the oxidation of unsaturated fatty acids by using Mn^3+^ as a mediator with the formation of peroxide radicals that will oxidize non-phenolic β-O-4-linked lignin (*17*, *21*). The presence of peroxide radicals of fatty acids in the oil causes an increase in its peroxide value, which is a marker of low oil quality.

The polysaccharide analysis of the hemp seed kernel detected unesterified and partially esterified homogalacturonan, and also low amounts of arabinogalactan proteins, rhamnogalacturonan I, callose, polysaccharides containing α-1,5-l-arabinan (*22*). Among the non-starch polysaccharides of soybeans 8 % cellulose and 17 % pectins were detected, which are cell wall constituents. The most abundant pectins were rhamnogalacturonans, arabinogalactan I and xylogalacturonan (*24*). Microscopic analysis of stained samples and fluorescence microscopy revealed the microstructure of oilseeds and oil-containing plant tissues (Table 1). Pectin-rich regions were detected by fluorescence staining with coriphosphine O, lignified cell walls were stained with Alcian blue, and the turquoise-blue colour represents the autofluorescence of protein bodies within the storage cotyledons.

The hemicellulose composition of plant cells is very complex. The monoclonal antibody analysis of hemicellulose composition of soybean seeds revealed galacturonan, homogalacturonan, xylogalacturonan, xyloglucan, mannan, galactan, arabinan and rhamnogalacturonan (*25*). The total polysaccharide composition of rapeseed includes 3.5 % arabinogalactan, 6.9 % arabinan, 15.5 % amyloid, 24.1 % cellulose and 50.0 % pectin, while the secondary cell walls of rapeseeds contain 39.0 % pectins, 29.0 % hemicelluloses, 22.0 % cellulose, 8.0 % of arabinogalactans and they make 20.0 to 28.0 % of the total seed (*18*, *37*). Rice bran dietary fibre contains approx. 90 % insoluble dietary fibre represented by cellulose, hemicellulose and arabinoxylans, and 10 % soluble dietary fibre, such as pectin and β-glucan. Feruloyl polysaccharide in the rice bran is represented by oryzanol, which is a mixture of ferulic acid esters of sterol and triterpene alcohols (*23*, *39*). The polysaccharide fraction of defatted *Moringa oleifera* seed flour consists of arabinogalactan, xylan-type polysaccharides and cellulose (*41*).

## ENZYMES DEGRADING POLYSACCHARIDES IN PLANT CELL WALLS

Despite the differences in the chemical composition of various types of oilseeds obtained in different studies, it can be concluded that the use of delignifying enzymes to destroy the cell walls of seeds in order to improve oil production may be inappropriate, because lignin is more abundant in the seed husks than in the oil-containing kernels, and the oxidases themselves pose risks to the obtained oil quality. Much more promising is the application of hydrolytic enzymes for cellulose and hemicellulose hydrolysis. The following enzymes have been used for this purpose (Table 1): exocellobiohydrolase (EC 3.2.1.91), endocellulase (EC 3.2.1.4), xylanase (EC 3.2.1.8), β-xylosidase (EC 3.2.1.37), β-mannanase (EC 3.2.1.78), β-mannosidase (EC 3.2.1.25), α-l-arabinofuranosidase (EC 3.2.1.55), α-l-arabinanase (EC 3.2.1.99), acetylxylan esterase (EC 3.1.1.72) and feruloyl esterase (EC 3.1.1.73). It is also possible to divide into two separate subgroups xyloglucan-degrading enzymes, such as xyloglucan-specific endo-β-1,4-glucanase (EC 3.2.1.151), xyloglucan-specific exo-β-1,4-glucanase (EC 3.2.1.155), oligoxyloglucan β-glycosidase (EC 3.2.1.120), and arabinogalactan-degrading enzymes, represented by galactan-1,3-β-galactosidase (EC 3.2.1.145), galactan endo-β-1,3-galactanase (EC 3.2.1.181), galactan endo-1,6-β-galactosidase (EC 3.2.1.164) and arabinogalactan endo-β-1,4-galactanase (EC 3.2.1.89). Because oilseed kernels also contain many pectin substances, including homogalacturonans and rhamnogalacturonans, the following enzymes have been used for their degradation: endopolymethylgalacturonate lyase (EC 4.2.2.10), exopolymethylgalacturonate lyase (EC 4.2.2.27), pectinesterase (EC 3.1.1.11), and rhamnogalacturonan-degrading enzymes, such as rhamnogalacturonan hydrolase (EC 3.2.1.171), rhamnogalacturonan galacturonohydrolase (EC 3.2.1.173), rhamnogalacturonan rhamnohydrolase (EC 3.2.1.174) and rhamnogalacturonan endolyase (EC 4.2.2.23) (Table 1).

## ENZYMES DEGRADING PROTEINS IN CELL WALLS AND LIPID BODY MEMEBRANES

The cotyledon is the main tissue in oilseeds, where proteins and lipids are accumulated and stored in the form of protein and lipid bodies. Lipid bodies in seed cells are woven into a cytoplasmic membrane consisting of proteins and also externally protected by a stable pectin and lignocellulosic matrix of seed cell walls (*12*, *13*). The epidermal cells of the *Moringa oleifera* seed cotyledon (image j in Table 1), as well as the rapeseed cotyledon cells (image d in Table 1), are occupied by lipid bodies, whereas protein bodies are seldom present and starch grains are totally absent (*36*, *40*). Subepidermal cells of the cotyledon contain lipid bodies surrounded by the protein bodies and they fill most of the remaining space in all healthy cells (*40*). Most of the rice bran lipids are in a form of lipid bodies in the aleurone layer (*23*). Based on the structure of lipid bodies to intensify their destruction and improve oil extraction from oilseeds, it is advisable to use proteases that destroy not only proteins of the oleosome membranes, but also glycoproteins in the cell walls and cytoplasmic membranes, including arabinogalactan proteins.

Among proteolytic enzymes, proteases of animal, plant, fungal and bacterial origin with broad specificity deserve attention (Table 2 (*58**-**96*)). Nowadays, various proteases are used by researchers to improve the production of protein products from natural sources, including oilseeds. It was proposed to prepare soy protein hydrolysates selectively enriched with the soybean protein glycinin and β-conglycinin by hydrolysis with pepsin and papain, respectively (*97*). Rapeseed protein isolate was hydrolyzed with Alcalase, Proteinase K, pepsin+pancreatin (P+P), thermolysin and Flavourzyme under different conditions (*98*). Porcine pepsin and pancreatin treatment were used to obtain sunflower protein hydrolysates (*99*). Bromelain and papain, which are used for tenderizing squid (*Loligo vulgaris*) muscle, are also potentially useful proteases (*58*). The hullless pumpkin oil cake protein isolate was hydrolysed by pepsin (*100*). In addition, the effectiveness of pepsin for the destruction of oleosomes has also been proven. Gastric digestion of a walnut oil bodies by pepsin was investigated *in vitro*. The results of the experiment revealed that such kind of treatment causes destabilization and coalescence of the oleosomes (*101*).

**Table 2:** Enzymes which can potentially be used for degradation of oilseed cell membranes and oleosome membranes

Enzyme	Origin/Producer	Substrate	Reaction product	Ref.
Proteases				
Serine proteases (Exopeptidases)				
Carboxypeptidase Y EC 3.4.16.5	*Saccharomyces cerevisiae*	Proteins with C-terminal side of the amino acids with broad specificity	Peptide and proteinogenic amino acid	(*60*)
Metalloproteases (Exopeptidases)				
Carboxypeptidase A EC 3.4.17.1	Bovine pancreas	Proteins with C-terminal side of the amino acids, but has little or no action Asp, Glu, Arg, Lys or Pro	Peptide and l-amino acid	(*60*)
Carboxypeptidase B EC 3.4.17.2	Porcine pancreas	Hydrolysis of proteins with C-terminal side of the Lys or Arg amino acids	Peptide and l-amino acid (l-Lys or l-Arg)	(*60*, *61*)
Serine proteases (Endopeptidases)				
Trypsin EC 3.4.21.4	Porcine pancreas	Protein at positions Arg-|-, Lys-|-	Peptide	(*60*-*63*)
Elastase EC 3.4.21.36	Porcine pancreas	Proteins, including elastin, at position Ala-|-	Peptide	(*60*, *61*)
Proteinase K EC 3.4.21.64	*Tritirachium album*	Peptide amides and proteins, including keratin	Peptide	(*60*, *64*)
Cysteine proteases (Endopeptidases)				
Papain EC 3.4.22.2	Latex of the papaya (*Carica papaya*) fruit	Proteins with broad specificity, especially an amino acid bearing a large hydrophobic side chain at the P2 position, and protein at positions Arg-|-, Lys-|-	Peptide	(*58,60,* *65,66)*
Stem bromelain EC 3.4.22.32	Stem of pineapples (*Ananas comosus*)	Proteins with broad specificity, but strong preference for Z-Arg-Arg-|-NHMec amongst small molecule substrates	Peptide	(*58,60,* *67,68)*
Fruit bromelain EC 3.4.22.33	Fruit of pineapples (*Ananas comosus*)	Proteins with broad specificity, especially the Bz-Phe-Val-Arg-|-NHMec links, but no action on Z-Arg-Arg-NHMec	Peptide	(*58,60*, *67,68)*
Ficin (ficain) EC 3.4.22.3	Latex of the fig (*Ficus carica*, *Ficus glabrata*)	Proteins with broad specificity, similar to that of papain	Peptide	(*60*, *69*)
Aspartic proteases (Endopeptidases)				
Pepsin (pepsin A) EC 3.4.23.1	Porcine gastric mucosa	Protein Phe (or Tyr, Leu, Trp)-|- Trp (or Phe, Tyr, Leu) links	Peptide	(*60*, *70*)
Aspergillopepsin I EC 3.4.23.18	*Aspergillus species*	Proteins with broad specificity	Peptide	(*71*)
Penicillopepsin EC 3.4.23.20	*Penicillium janthinellum*	Proteins with broad specificity similar to that of pepsin A, preferring hydrophobic residues	Peptide	(*71*)
Rhizopuspepsin EC 3.4.23.21	*Rhizopus chinensis,* *R. niveus*	Proteins with broad specificity, prefers hydrophobic residues, clots milk, and activates trypsinogen	Peptide	(*71*)
Saccharopepsin EC 3.4.23.25	*Saccharomyces cerevisiae*	Proteins with broad specificity for peptide bonds	Peptide	(*71*)
Metalloproteases (Endopeptidases)				
Thermolysin EC 3.4.24.27	*Bacillus thermoproteolyticus*	Protein Leu (or Phe)-|- Leu (or Phe, Val, Met, Ala, Ile) links	Peptide	(*60*, *72*, *73*)
Phospholipases				
Phospholipase A_1_ EC 3.1.1.32	*Aspergillus oryzae*	Broad specificity, but prefers phosphatidyl choline	Lysophosphatidyl choline and fatty acid	(*74*, *75*)
Phospholipase A_2_ EC 3.1.1.4	Honey bee venom (*Apis mellifera*), bovine pancreas	Ester linkage at *sn-*2 carbon of fatty acid acyl bond of phospholipids, preferring phospholipids containing arachidonic acid	Lysophospholipid and free fatty acid	(*74*, *76*-*79*)
Phospholipase C EC 3.1.4.3	*Bacillus cereus, Clostridium perfringens*	The bond between the acylglycerol and the phosphate group, prefers phosphatidyl choline, phosphatidyl ethanolamine, sphingomyelin and phosphatidyl inositol	1,2-Diacyl-*sn*-glycerol and phosphate monoester	(*74*, *75*, *80**-**83*)
Phospholipase D EC 3.1.4.4	*Streptomyces* sp., peanut (*Arachis hypogaea*)	Phosphate diester bond of glycerophosphatides containing choline, ethanolamine, serine, or glycerol, preferring phosphatidyl choline	Phosphatidic acid and choline	(*74*, *84*, *85*)
Phosphatases				
Alkaline phosphatase EC 3.1.3.1	*Escherichia coli,* bovine intestinal mucosa	Phosphate monoester	Alcohol and phosphate	(*59*, *86*-*88*)
Protein phosphatase EC 3.1.3.16	*Escherichia coli*, bovine kidneys, bovine brain	Serine- or threonine-bound phosphate group from a wide range of phosphoproteins	Protein (containing Ser/Thr) and phosphate	(*59*, *89*-*91*)
Acid phosphatase EC 3.1.3.2	Lupin seeds (*Lupinus luteus*), potato tubers	Phosphate monoester	Alcohol and phosphate	(*59*, *92*)
Phosphatidate phosphatase EC 3.1.3.4	Cotton seed (*Gossypium hirsutum* L.), *Saccharomyces cerevisiae*	1,2-Diacyl-*sn*-glycerol-3-phosphate	1,2-Diacyl-*sn*-glycerol and phosphate	(*59*, *93*-*96*)

Despite the beneficial effect of proteases on the oil yield, it is important to remember that some preparations such as pancreatin contain not only various proteases and amylases, but also lipases, which catalyze the hydrolysis of triacylglycerols (*59*). Such an effect is positive for the production of protein isolates or protein hydrolysates since it allows to increase product purity, but the presence of lipases for oil production is highly undesirable because it promotes the destruction of lipids and the release of free fatty acids (FFA). An increased amount of FFA in oil is a marker of reduced quality. Therefore, during the enzymatic treatment of oilseeds and oil-containing plant materials, it would be appropriate to use a combination of pepsin with another pancreatic enzyme like trypsin (EC 3.4.21.4) or elastase (EC 3.4.21.36) instead of pancreatin.

## ENZYMES DEGRADING PHOSPHOLIPID MEMEBRANES

The destruction of the phospholipid component of oleosome membranes is facilitated by the use of phospholipases and phosphatases. Phosphatases and phospholipases are very common in nature and regulate metabolic processes in animal, plant and microorganism cells. Phospholipases hydrolyze phospholipids and release lysophospholipids, free fatty acids, diacylglycerols, choline phosphate and phosphatidates, while phosphatases catalyze the hydrolysis of phosphomonoesters (*59*, *74*-*96*, *102*, *103*). Traditionally, phospholipases are used in the industry for degumming of vegetable oils, as a replacement for emulsifiers in bread making to increase the dough stability, in dairy to increase cheese yield by hydrolysis of milk phospholipids, and for the modification of egg yolk in mayonnaise preparation (*74*). Most phospholipases catalyze the hydrolysis of phosphatidyl choline (Table 2), which, as mentioned above, predominates in the phospholipid layer of oleosomes. Phospholipids are also important components of all cell membranes (*14*, *15*, *74*-*85*). It was found that the destruction of phospholipids by phosphatases detected in oilseeds causes the destruction of cell membranes. Protein bodies of the *Moringa oleifera* seed cells contain several hydrolytic enzymes and phosphatases and their release into the cytosol causes localized cellular autolysis and membrane deterioration (*40*). Therefore, the prospect of possible use of phospholipases and phosphatases for the destruction of seed cell membranes and oil body membranes could be a strategy to increase the oil yield during pressing.

At the same time, this enzymatic treatment should be used with caution. Authors who studied the effect of phospholipases in the degumming of vegetable oils on their quality reported an increase in the peroxide value of sunflower oil after treatment with phospholipase A_1_, and a decrease in oxidative stability of rapeseed oil after degumming with phospholipase C and soybean oil with each of phospholipases A_1_ and C (*80*-*82*). Such changes can probably be explained by the fact that the destroyed phospholipids could play the role of natural antioxidants in oils (*81*, *82*).

## COMBINATION OF ENZYMES USED FOR OILSEED PRETREATMENT

Given the multicomponent composition of oilseed cells, it can be deduced that for a maximum oil release it is important to destruct the polysaccharides which are cell wall components together with proteins and possibly also phospholipids that are components of the cytoplasmic membrane and oleosome membranes. For such a strategy a mixture of enzymes with different activities is suggested. For example, enzymes used to hydrolize plant protein isolates contain a combination of proteases (brand names) like Alcalase (*Bacillus licheniformis*, P4860), Neutrase (*Bacillus amyloliquefaciens*, P1236) and Flavourzyme (*Aspergillus oryzae*, P6110) (*104*). For the production of protein isolates from pumpkin seeds, a mixture of pectinase and cellulase in a ratio of 1:1 was employed (*105*) and for the extraction of polysaccharides from bamboo shoots or ginger stems, an enzyme mixture of cellulase, papain and pectinase in a ratio of 1:1:1 worked well (*106*, *107*).

The use of enzymes with different activities has also been investigated for the extraction of vegetable oils from seeds, *e.g.* a mixture of proteases and cellulases in different ratios and under different conditions in order to increase the yield of pressed oil from pumpkin seed and rapeseed (*108*-*110*). Many researchers have treated various oilseeds with mixtures consisting of enzymes with proteolytic, cellulolytic and pectolytic activity (*6*, *9*, *13*, *111*, *112*). Some studies have also used the treatment of oil material with separate enzyme preparations, such as Ronozyme VP (endoglucanase, hemicellulase and pectinase) from DSM Nutritional Products (Basel, Switzerland), Protex 7L (protease) and Multifect CX 13L (cellulase, β-glucanase, hemicellulase and arabinoxylanase) from Genencor (Rochester, NY, USA), Viscozyme L (cellulase, β-glucanase, hemicellulase, arabanase and xylanase) from Novozymes (Bagsvaerd, Denmark), Natuzyme (cellulase, α-amylase, pectinase, xylanase and phytase) from Bioproton Pty Ltd (Acacia Ridge, Australia), and Kemzyme (protease, cellulase, β-glucanase, α-amylase, hemicellulase and xylanase) from Kemin Europa N.V. (Herentals, Belgium) (*11*, *25*, *113*, *114*). *In vitro* non-starch polysaccharide analyses of solubilized soybean meal cell walls after treatment with the multienzyme product Ronozyme VP (*Aspergillus aculeatus*) revealed a statistically significant reduction in insoluble sugar residues for (in %): rhamnose 35, arabinose 36, galactose 36 and glucose 39. The solubilisation of xylose, mannose and uronic acid was about 18, 14 and 22 % respectively. The specific degradation of pectin homogalacturonan epitopes, β-1,4-mannan, xyloglucan, galactan and arabinan of soybean meal cell walls was detected with monoclonal antibodies (*25*). The treatment of *Moringa oleifera* seed with Protex 7L increased the oil yield by 69.4 % compared to all other enzymes, which can be explained by its proteolytic activity and, consequently, by hydrolysis of proteins that are part of lipid spherosome membranes, unlike other enzymes, which were characterized by more cellulolytic and pectolytic activity (*114*).

## FACTORS INFLUENCING ENZYME ACTIVITY AND EFFICIENCY

### Particle size

It is known that increasing the degree of grinding of the material particles leads to additional destruction of its cell walls, thereby increasing the yield of the finished product (*7*, *12*, *18*, *115*). At the same time, the smaller the particle size of the material, the larger its surface area in contact with enzymes (*12*, *115*, *116*). The degree of grinding is an important parameter that affects the yield of oil from the oil material, both with and without enzymatic pretreatment (*18*). A study on grape seeds using solvent extraction in the Soxhlet apparatus without prior enzymatic treatment found that reducing the particle size from 1.0–1.4 to ˂0.5 mm leads to an increase in oil extraction yield from 6.66 to 15.30 %, while at the particle sizes of 1.0–1.4 mm with enzymatic pretreatment, the extraction yield ranged from 6.71 to 17.5 %, and the highest oil yield of 19.5 % was achieved after fermentation of the material with particle sizes ˂0.5 mm (*112*). By reducing the particle size of soybean seeds from 2.5 to 0.5 mm during the solvent extraction, the extractability (expressed as the reduction of oil relative to the total oil content of the material) of prefermented samples increased from 25 to 60 %, while the extractability of untreated samples increased from 15 to 45 %, respectively (*117*). However, it is also important to consider that excessive reduction of the particle size of the oleaginous material during extraction with an organic solvent can lead to particle adhesion, microporosity reduction of the material and, as a result, it prevents the solvent movement between particles and decreases the oil yield (*116*).

Very small particle size of oilseeds is also undesirable for enzyme-assisted aqueous extraction (EAAE), as well as for solvent extraction. The demulsification process might become more complicated due to the reduction in the oleosome sizes caused by excessive grinding of the material (*12*, *115*). The particle sizes of sunflower seeds were 0.762–1.0 mm for enzyme-assisted hexane extraction (EAHE) and EAAE. Oil yields ranged from 41.36 to 55.38 % with EAHE and from 15.95 to 34.05 % with EAAE, depending on the method of seed preparation for extraction (*118*). During the ultrasound-assisted aqueous enzymatic extraction, the particle size of the perilla seeds was 0.8–1.2 mm, the highest oil yield reached 32.66 % (calculated on seed mass basis), while in another study of the ultrasonic-assisted aqueous enzymatic extraction of perilla seeds with a particle size of 250 µm, the maximum oil yield was 31.47 % (*111*, *113*). Cotton, hemp, sunflower, sesame, canola and *Moringa oleifera* seeds were ground for EAAE to a fraction passing through an 80-mesh sieve (mesh size of 0.177 mm), oil yields ranged from: 3.0 to 6.5, 22.5 to 29.0, 24.0 to 40.0, 13.0 to 25.0, 19.5 to 26.0, and 18.5 to 22.5 %, respectively, depending on the type of enzyme used to treat the seeds and other parameters of the fermentation process, such as pH, enzyme concentration, water/seed ratio or moisture content, temperature and time of extraction. The same particle size of oleaginous material was also used in the study of oil extraction from cotton, hemp, sunflower, sesame, canola and *Moringa oleifera* seeds by enzyme-assisted cold pressing (EACP). The oil yields varied from: 5.5 to 13.0, 25.7 to 32.7, 31.5 to 39.0, 23.7 to 28.1, 23.0 to 28.5 and 15.5 to 21.5 %, respectively (*11*). Soybean seed flakes with particle sizes of 0.5–1.0 mm and soybean seed collets with an average diameter of 20 mm and a length of 50 to 100 mm were subjected to enzyme-assisted mechanical pressing. Pressing of flakes at 65 MPa allowed to obtain oil yield from 7.91 to 12.3 % (calculated on seed mass basis), while during pressing of collets at pressures of 37 and 65 MPa oil yield varied from 8.0 to 11.96 % and 14.34 to 18.75 %, respectively, depending on the enzyme used to treat the oleaginous material (*119*). During the pressing of rice bran crushed to a fraction passing through a 20-mesh sieve (mesh size of 0.841 mm), the yield of the obtained oil was 16.5 % (*6*).

Therefore, during the enzyme-assisted pressing, as well as at the enzyme-assisted aqueous extraction, reduction of the seed particle sizes within a certain interval helps to increase oil yield; however, excessive grinding is inefficient, so this parameter has to be optimized for each case.

### Amount of added water

Water is a necessary component to enable enzymatic activity and perform hydrolysis. However, its amount in relation to the substrate is limited by the used extraction technology. In particular, during aqueous enzymatic extraction, which is commonly used to obtain protein hydrolysates and protein isolates from vegetable raw materials, the substrate/water mass ratio can be quite low, *e.g.* 1:6, 1:10, 1:20, 1:25, 1:40 and 1:100 (*97*-*99*, *120*-*127*). Also, for the extraction of polysaccharides, high water mass ratios ranging from 1:20 to 1:100 have been reported (*106*, *107*, *128*). Such a large amount of added water can be explained by the fact that proteins and polysaccharides are hydrophilic and more water is beneficial for an efficient solubilization and extraction from vegetable raw materials. For the extraction of oil from the various oilseeds after enzymatic treatment, much lower water mass ratios were applied. Perilla seeds were treated with separate enzyme preparations Cellulase, Viscozyme L, Alcalase 2.4 L, Protex 6 L, and Protex 7 L at a substrate/water mass ratio of 1:6, as well as a mixture of cellulase, neutral proteinase and pectinase at a substrate/water mass ratio of 1:3 to 1:7. An oil yield of 31.34 % was obtained at a mass ratio of 1:4.4, which was chosen as the optimal for this oil crop (*111*, *113*). Aqueous enzymatic extraction of sunflower kernels was performed using Viscozyme L at a substrate/water mass ratio of 1:6. The highest oil yield of 34.05 % (which represented 61.46 % of the total extractable oil) was obtained from raw sunflower kernels by this kind of treatment (*118*).

Despite the advantages and significant number of studies on aqueous enzymatic extraction, this method has not found wide practical application, in particular for the extraction of vegetable oils. Nowadays, most of vegetable oil producers use either pressing, or solvent extraction, or a combination of both. A significant disadvantage of aqueous enzymatic extraction is the formation of an emulsion, which is then difficult to separate (*7*).

**Fig.1:**
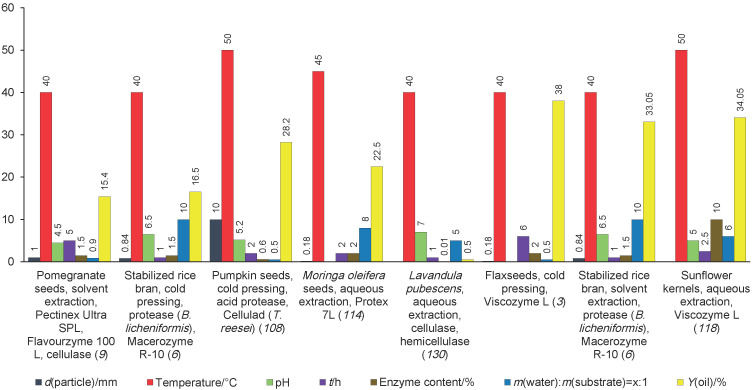
Parameters of enzymatic hydrolysis of oilseeds and oil yields. References are given in brackets

Many researchers have worked to combine the hydrolytic enzyme pretreatment of oil material with methods that are widely used in the industry. Different mixtures of enzymes with proteolytic, cellulolytic and pectolytic activity were used at substrate/water mass ratios of 1:5.5; 1:7 and 1:10.5 for enzymatic pretreatment of cotton seeds with subsequent hexane extraction of oil from the treated material (*129*). However, to combine enzymatic pretreatment with the oil extraction method, the use of water amounts that exceed several times the substrate amount is highly undesirable. Applying excessive amounts of water will significantly increase the cost of time and energy for the material drying to bring it to the required value of moisture for pressing, the process will be technologically more complicated and economically unprofitable (*11*). In addition, a prolonged contact of oil material with free moisture can cause hydrolysis of oil, which can further adversely affect its quality after removal from the seed. Most scientists who developed the technology of enzymatic treatment before pressing of various oilseeds tried to minimize the amount of water added to the substrate during enzymatic hydrolysis (Fig. 1 (*3*, *6*, *9*, *108*, *114*, *118*, *130*)), in particular, they reduced the substrate/water mass ratio to 1:1, or to the amount of water relative to substrate 45–50 % (*3*, *4*, *9*, *10*, *108*-*110*, *119*). There are also studies where the preliminary enzymatic treatment of oil material was performed at different water amount added to the substrate. The enzymatic pretreatment of apricot kernels with pectolytic and cellulolytic enzymes was investigated at 20 to 32 % water added during hydrolysis. The highest value of increased oil recovery (2.53 %) was achieved when 23 % of water was added (on substrate mass basis) (*131*). The enzymatic hydrolysis of borage seeds with enzyme preparations Olivex and Celluclast was performed using 20 to 50 % of water. Better result (oil yield of 85.5 %) was obtained with 20 % of water (*132*). Rape, sunflower, sesame, cotton, hemp and *Moringa oleifera* seeds were enzymatically pretreated using 35 to 55 % of water. It was found that the addition of water between 35 and 45 % was optimal for most of these crops during hydrolysis (*11*).

### Enzyme to substrate ratio

The mass ratio of enzyme to substrate during hydrolysis may vary depending on the enzyme activity, the substrate nature, as well as the desirable depth of hydrolysis. Alcalase, Proteinase K, pepsin, pancreatin, thermolysin and Flavourzyme were used to obtain protein hydrolysates of rapeseed and oat bran at *w*(enzyme)=4 % and *w*(pepsin+pancreatin)=4.0 %. The highest yield of rapeseed protein hydrolysate (76.67 %) was produced by Alcalase, and the lowest yield (36.18 %) was produced by Flavourzyme (*98*, *121*). A mixture of *w*(pepsin+pancreatin)=10 % was used to obtain protein hydrolysates from low-fat *Jatropha curcas* flour. The degree of hydrolyzation of protein hydrolysates obtained from defatted *Jatropha curcas* flour was 19.3, 18.8 and 19.0 % at 60, 90 and 120 min, respectively (*123*).

It is known that increasing the enzyme amount increases the volumetric activity; however, this is only valid until the enzyme fully saturates the substrate surface, after that increasing the amount of enzyme will no longer be effective (*7*). As for the use of enzymatic pretreatment of oil material before pressing, an excessive increase of the enzyme amount will not be economically profitable due to its high cost. Excess enzyme can also lead to hydrolysis of polysaccharides and to the formation of free reducing sugars, which will caramelize during the drying of oil-containing plant material immediately before pressing, interfere with oil production and reduce the oil yield (*7*, *11*). In addition, excessive increases of the enzyme amount can lead to deterioration of the obtained product by causing odours and bitterness (*7*). That is why it is important to find the optimal amount of individual enzymes or their mixtures for each type of substrate, which is sufficient to ensure maximum efficiency, but does not lead to the formation of undesirable by-products.

Studies testing enzymatic pretreatment of various oilseeds performed at an enzyme mass fraction of 2.0–2.5 % (*3*, *10*, *133*) showed that such an enzyme amount was optimal during the optimization of enzymatic hydrolysis parameters (*6*, *11*). Aqueous enzymatic extraction of pumpkin seed oil was performed by using a mixture of proteolytic, cellulolytic and pectolytic enzymes at the amount of 2.0 % by mass of seeds. The oil recovery ranged from 37.81 to 72.91 % (*13*).

The work of other researchers has also been devoted to the study of the effect of the amount of enzymes or their mixtures on the oil yield after enzymatic pretreatment of seeds. Aqueous enzymatic extraction of oil from the palm pulp was performed by using a mixture in the range from 0 to 1.0 % of enzyme preparations Cellic CTec2, Cellic HTec2 and Pectinex Ultra SP-LThe highest oil yield of 88.0 % was obtained at the optimal amounts of these enzyme preparations taken in the mass ratio of 0.46:0.34:0.2 %, respectively (*134*). It was found that the optimal amount of enzyme mixtures with cellulolytic and pectolytic activity for enzymatic hydrolysis of soybean seeds is 1.0 %, and for sunflower seeds 2.0 %, with achieved oil extractability of 55.0 and 98.5 %, respectively (*117*). The enzymatic pretreatment of rapeseed was carried out by using a mixture of enzyme preparations Protolad and Celulad at the amount from 0.4 to 1.4 % by mass of the substrate. The optimal amount of enzymes was 0.4 %, the oil yield ranged from 32.2 to 45.9 %, depending on other process parameters, such as processing time and moisture content before pressing (*109*, *110*).

There are also studies where enzymatic pretreatment of borage, soybean and pumpkin seeds was carried out by using the enzyme preparations of 0.25, 0.5 and 0.6 % by mass of seeds, respectively (*108*, *119*, *132*).

### Process pH

Enzymatic activity depends on the pH of their environment. Each enzyme has an optimal range of pH in which it shows maximal activity, but also the stability of the enzyme depends on pH (*7*, *135*, *136*). The effect of the pH on enzyme activity primarily originates from ionizable amino acids in the catalytic site as well as in the binding site, which influences the formation of the enzyme-substrate complex and the activation of the substrate (*135*, *136*). The pea protein isolates were hydrolyzed by using Alcalase, Neutrase and Flavorzyme, as well as mixtures thereof at pH=7.4 (*104*). Protein isolates of oat bran were prepared by hydrolysis with cellulase or Viscozyme L at pH=4.5 for 1.5 h, then pH was raised to 9.5 and the process continued for another 1.5 h to dissolve the proteins. The soluble protein content of extracted proteins from Viscozyme-treated oat brans was (86.3±3.3) % on a dry mass basis, compared to the value of (51.4±2.4) % obtained from cellulase-treated brans (*121*). For the production of protein hydrolysates, low-fat *Jatropha curcas* flour was first hydrolysed with Alcalase at pH=8.0 for 1.0–2.0 h, then for 0.5–1.0 h with pepsin at pH=2.0 and again for 0.5–1.0 h with pancreatin at pH=7.5. The degree of hydrolyzation of the protein hydrolysates obtained from defatted *J. curcas* flour with Alcalase was 10.4, 10.6 and 11.5 %, while 19.3, 18.8 and 19.0 % was achieved with pepsin–pancreatin at 60, 90 and 120 min, respectively (*123*). The pumpkin flour was hydrolyzed in the presence of cellulase at a pH=3.0 to 5.5 to obtain water-soluble polysaccharides. The highest yield of polysaccharides (17.34 %) was observed at pH=4.5 (*128*). Sulfated polysaccharides were removed from green seaweed *Ulva lactuca* by hydrolysis first using cellulase (C-2730) with an activity of 700 U/g at pH=5.0, and then protease (P-1236) with an activity of 0.8 U/g at pH=7.0, which led to the highest yield of 17.14 % polysaccharides, while the lowest yield of 3.04 % was obtained by hydrolysis without enzymes at pH=1.5 and 90 °C (*137*).

It was found that for many oilseeds the results of the oil and protein aqueous extraction correlate: the process conditions resulting in the highest oil yield often also coincide with the conditions resulting in the highest protein yield. The authors suggest that the basis of this pattern is the dependence of the protein solubility on the pH value. During aqueous extraction the highest oil yield is obtained at pH values ​​corresponding to the maximum solubility of the protein in the aqueous system, and the lowest oil yield is obtained when the protein solubility is the lowest, supposedly at the isoelectric point. At the pH range corresponding to the isoelectric point, the protein binds the oil much better, which prevents its release, so for most oil seeds aqueous extraction of oil and proteins is tried at pH values not close to the isoelectric point. The total isoelectric point of most oilseed proteins usually corresponds to a pH=4.0–5.0 (*7*, *115*).

Aqueous enzymatic extraction of essential oil from *Lavandula pubescens* was performed using enzymes with cellulolytic activity at pH=7.0. The highest essential oil yield of 0.50 % was achieved by cellulase pretreatment (*130*). Aqueous enzymatic extraction of perilla seeds was carried out with an enzymatic mixture of cellulase, neutral proteinase and pectinase also at pH=7.0. The oil yield ranged from 24.13 to 31.47 %, depending on other process parameters, such as liquid-to-solid ratio, hydrolysis time, hydrolysis temperature and ultrasound treatment time (*111*). Some researchers also enzymatically pretreated soybean, sunflower, rape and borage seeds by using proteolytic, cellulolytic and pectolytic enzyme preparations and their mixtures at a pH value corresponding to the pH of distilled water (6.5–7.0) (Fig. 1) (*109*, *110*, *117*, *132*).

It is also necessary to consider the pH range of each enzyme in enzyme mixtures. Combining enzymes with similar pH ranges together allows their effective use in a single step process and avoids the necessity to separate the hydrolysis process in two or more stages. The pH optimum for most enzymes with cellulolytic and pectolytic activities is in the range of pH=​​4.5–5.5. Enzymatic pretreatment with the commercial enzyme preparations Kemzyme, Feedzyme, Natuzyme, Phytezyme, Allzyme and Viscozyme L of rapeseed, sunflower, sesame, cotton, hemp and *Moringa oifera* has shown that pH=5.0 is optimal (*11*). EAAE of pumpkin seed oil was performed using mixtures of enzyme preparations with proteolytic, cellulolytic and pectolytic activities at pH ranging from 4.0 to 5.0. The highest oil yield of 72.64 % was obtained at pH=4.7 (*13*). An enzymatic mixture of cellulase and pectinase at pH=5.0 was used during aqueous enzymatic extraction of pumpkin seed proteins. The highest yield of protein isolate was 9 g of soluble protein from 100 g pumpkin seeds (*105*). Many studies (Fig. 1) have also used proteolytic, cellulolytic and pectolytic enzyme preparations at pH ranging from 4.5 to 5.0 during enzymatic pretreatment of other oilseeds (*6*, *9*, *108*, *133*, *134*).

### Temperature of the enzymatic hydrolysis

Thermostability is another important characteristic of enzymes, which determines their applicability in industrial processes. High temperatures are beneficial for hydrolytic processes, because the rate of the reaction increases with the temperature. This is also true for enzymatic reactions; however, the protein fold of enzymes limits the maximal applicable temperature. The catalytic activity of most enzymes increases up to 50 °C; above this temperature the denaturation of the enzyme starts and reduces activity by irreversible denaturation (*135*, *136*, *138*). However, despite the general pattern of enzymatic activity dependence on temperature regimes, each individual enzyme has an individual temperature optimum, according to the type of enzyme, protein fold and also its substrate.

An example is the production of protein hydrolysates from different plant raw materials for which a combination of the natural enzymes pepsin and pancreatin was used at a hydrolysis temperature of 37 °C (*98*, *99*, *120*-*123*). At the same time, the thermostable, engineered proteases Alcalase and Flavourzyme were used without detrimental effects on the enzyme activity at 50–60 °C (*98*, *121*, *123*). The enzymatic hydrolysis of biogas residues was performed using cellulase at 50 °C (*139*). The enzymatic saccharification of bamboo residues was performed at the same temperature with a mixture consisting of xylanase, α-l-arabinofuranosidase and cellulase. The xylan degradation yield of the sample pulped with 12 % effective alkali charge increased from 68.20 to 88.35 %, while the enzymatic saccharification efficiency increased from 58.98 to 83.23 % (*140*). Water-soluble polysaccharides were obtained from pumpkin flour by hydrolysis using cellulase at 40 to 65 °C. The highest yield of polysaccharides (17.34 %) was achieved at 55 °C (*128*). The enzymatic pretreatment of cotton seeds was performed with individual enzymes, in particular papain at 25 °C, bacterial protease at 37 °C, Savinase at 55–60 °C and Termamyl at 85–115 °C; the increase in oil extractability ranged from 4.32 to 27.73 % compared to control sample, depending also on other process parameters, such as enzyme amount, time and water/substrate mass ratio. The enzyme mixtures used were: Savinase+bacterial protease at 40 °C, Savinase+papain at 30 °C, Savinase+Termamyl at 70 °C, Savinase+cellulase at 45 °C, Savinase+pectinase at 35 °C, Savinase+pectinase+bacterial protease at 50 °C and Savinase+pectinase+cellulase at 50 °C, and the relative increases in hexane-extracted oil were 37.1, 28.9, 34.9, 30.1, 39.7, 44.9 and 38.9 %, respectively (*129*).

At the same time, it was found that during the extraction of oil from different types of oilseeds, the highest oil yields were obtained at temperatures ranging from 40 to 60 °C (*12*, *115*). Enzymatic hydrolysis was carried out mainly at 40–50 °C in the studies (Fig. 1) using enzymes with proteolytic, cellulolytic and pectolytic activity to improve aqueous extraction, organic solvent extraction and press extraction of the oil (*3*, *4*, *6*, *9*, *10*, *109*-*114*, *117*, *118*, *130*, *131*, *133*, *134*).

During the production of pumpkin seed protein isolates and pumpkin seed oil, the temperature of the hydrolytic enzymatic treatment was 45 and 48–54 °C, resulting in the highest soluble protein content in protein isolate, and the oil yield increased from 62.3 % (control sample) to 70.0 % from total oil content (42.4 %) of seeds, respectively (*105*, *108*). Aqueous enzymatic extraction of pumpkin seed oil was performed by using a mixture of proteolytic, cellulolytic and pectolytic enzyme preparations at temperatures ranging from 45 to 55 °C. It was found that the optimal temperature for this process is 54 °C with achieved oil yield of 72.64 % (*13*).

### Duration of hydrolysis

A factor affecting the efficiency of enzymatic hydrolysis is the process time during which the enzyme can act on its substrate and weaken cellular structures. It is known that increasing the duration of enzymatic pretreatment of oilseeds to a certain point helps to increase oil yield (*11*). Soybean and sunflower seeds were treated with mixtures of cellulolytic and pectolytic enzymes for 1 to 12 h before removing the oil from the seeds by extraction with an organic solvent in a Soxhlet apparatus. It was found that the optimal duration of enzymatic hydrolysis of both types of seeds is 6 h, in which the extracted oil from soybean was 54.0 % and from sunflower seeds 97.8 %. A further increase of the process time did not increase the oil yield (*117*). This indicates that the cellular structures are sufficiently weakened after a certain time to be processed. At the same time, the content of free reducing sugars in the fermented material increases with the prolonging of hydrolysis time, which in further processing can caramelize and prevent the oil release. The excessive increase of the contact time of the oil-containing plant material with the aqueous enzyme solution can lead to the hydrolysis of triacylglycerols and the deterioration of the organoleptic product properties. A long process of preparing oil material for oil extraction can also reduce the economic profitability (*11*, *117*).

To increase the yield of olive oil, olive paste was treated with enzymatic mixtures of pectinase, cellulase and hemicellulose and the duration of the process varied from 0.5 to 2.5 h. Enzymatic treatment for 1.5 h was optimal for this type of oil material, resulting the oil yield of 15.72 g from 100 g paste (*141*). The treatment of rapeseeds before pressing was performed with a mixture of the commercial enzyme preparations Protolad and Cellulad for 2.0 to 4.0 h. As the study showed, the duration of the enzymatic treatment of oilseeds for 2 h is sufficient to obtain a high oil yield of 43.4 % (*109*, *110*). To increase oil yield from perilla seeds, pumpkin seeds, *Moringa oleifera* seeds and palm pulp, the plant material was treated with proteolytic, cellulolytic and pectolytic enzyme preparations for 2 h (Fig. 1) (*108*, *113*, *114*, *134*). The analysis of the data presented in Fig. 1 shows that the individually optimized factors for oil extraction are not valid for different oilseeds. However, the following trend is observed: a higher enzyme amount, a longer incubation time and a smaller particle size increase the oil yield.

## TECHNICAL CONSIDERATIONS FOR ENZYMATIC PRETREATMENT

The technology of oilseed preparation for oil extraction by pressing includes operations of seed reception, cleaning and weighing, dehulling, flaking and cooking (*142*, *143*). All these operations affect or are affected by enzymatic pretreatment. To prevent spoilage of seeds during long-term storage in the silos, as well as to facilitate their further technological processing and to obtain a high-quality product, seeds should be cleaned (*143*). Dehulling is the next operation after cleaning; this process usually consists of two stages: opening the seed coats by cracking and separating the hulls from the kernels by screening and aspiration. The presence of a certain amount of hulls in the oleaginous material is necessary to ensure its desired structure during pressing. However, the hull material is also a substrate for many of the reported hydrolases and would be a competitive substrate to the oilseed cells, thus reducing the enzymatic action on the oilseed cells. An approximate hull content of 8.0 % is recommended for sunflower kernels that undergo flaking and further processing. However, for some types of oilseeds that are very small in size and have the seed hulls firmly stuck to the kernels, the process of dehulling is impractical. For example, flaxseed and rapeseed are flaked immediately after cleaning (*142*, *143*). Small particles of oil-rich material will stick together and decrease the porosity. Therefore, the particle size after grinding should be optimal (*142*). The analysis of works devoted to the application of enzymatic treatment of oil-containing material before oil extraction showed that the process is effective if the particle size of the oilseeds is in the range of 0.5–1.0 mm (*6*, *11*, *111*-*113*, *117*-*119*). At the same time, according to the recommendations of the traditional technology of oil extraction from oleaginous material by pressing, the particle size should be 0.4–1.0 mm for sunflower seeds, approx. 1.0 mm for flax- and cottonseeds, and 0.25–0.30 mm for soybean seeds (*142*, *143*). Therefore, the particle size of the seeds in the range of 0.3–1.0 mm can be considered optimal for enzyme-assisted mechanical pressing, smaller diameters result in a higher specific surface area and provide a better accessibility of the plant structures by enzymes.

Cooking is a key step in preparing oilseeds for oil extraction by pressing. This process involves the simultaneous or sequential treatment of oleaginous material with water and heat in order to change its colloid-chemical and physico-mechanical properties, and to weaken the cell walls. Cooking is also a necessary operation for inactivating enzymatic activity, preventing the hydrolysis of triacylglycerols and development of mould and bacteria, as well as for binding anti-food substances, such as gossypol in cottonseeds (*142*-*145*).

Oilseeds contain their own set of enzymes that catalyze complex biochemical processes during storage and processing, which will affect the quality of the obtained oil. Lipases from oilseeds cause hydrolysis of triacylglycerols, accompanied by an increase in free fatty acids in the oil (*142*-*145*). In rice bran, lipase is so active that in the first hours of storage the acidity of crude oil increases at a rate of 1 % per h, 5–7 % per day, and can finally reach up to 50 % of free fatty acids (*143*, *146*). Flaxseeds, in addition to lipase, also contain the glucoside linamarin and the enzyme linase, which catalyzes the cleavage of its glucosidic bonds with the release of hydrocyanic acid. Press cake containing hydrocyanic acid can be dangerous to livestock during feeding (*142*, *143*). The effect of high humidity and temperature of 40–60 °C in the process of cooking will significantly increase the activity of these enzymes, while their inactivation requires short-term (30–40 s) heating of wet oilseeds at 80–90 °C. In industry, inactivation is carried out at the first stage of cooking in a separate screw steamer, or in the roaster, where all subsequent stages of cooking take place (*142*, *144*, *145*). It is recommended to sterilize rice bran immediately upon arrival at the plant by heating at 90–100 °C and drying to stop lipase activity, or to stabilize it by heating at 125–135 °C for 1–3 s at 11–15 % moisture or by wet extrusion at 120 °C and 10 % added water as steam (*143*, *146*).

During cooking, the hydrophilic gel particles increase significantly in volume, and as a result, the volume of all oil-filled cavities decreases, and the oil is pushed to the surface of the particles under the action of swelling pressure (*142*, *145*).

At the moistening stage during cooking in the industry, the oil-containing material is usually adjusted to the humidity values of 8.0–9.0 % for sunflower seeds and flaxseeds and 11.5–17.5 % for cottonseeds (*142*, *144*, *145*). However, for enzymatic pretreatment of oilseeds, the optimal amount of added water should be in the range of 20–50 % (*3*, *4*, *9*-*11*, *108*-*110*, *119*, *131*, *132*). A water content below 20 % may not be enough to fully carry out the enzymatic hydrolysis and to evenly distribute the dissolved enzyme between the particles of the material, while increasing the water level above 50 % will cause an increase of time and energy during drying and roasting. Under industrial conditions, the amount of water added to the oleaginous material during cooking is usually much smaller than the theoretically possible amount that can be absorbed and bound. For example, the maximum swelling of ground sunflower seed kernels is achieved at 35 % of the added water, which will be completely absorbed and bound. When the oil-containing material is overwetted, the processes of oil displacement by water become much more intense, due to swelling, which allows oil to be easily separated from the material even with very little external pressure (*142*). Already in 1929–1934, a method was invented to prepare oilseeds for oil extraction, which involves treating ground seeds with water or hot steam for 2–7 min at a temperature of 20–80 °C with bringing the oil-containing material to a humidity of 12–20 %. The method allowed to extract 60–70 % of high-quality oil from seeds; however, its disadvantages were the difficulty of introduction into continuous process and the high degree of denaturation of protein compounds in the material (*142*, *147*). Although this method has not become widespread in industry and has been quickly replaced by other technologies currently used in oil production, its basic idea allows us to make the assumption that adding more water (20–50 %) to the oleaginous material during enzymatic pretreatment may be acceptable for cooking.

The wet material is excessively ductile, which makes it impossible to use for pressing. To remove excess moisture from the oil-containing material and provide a more rigid structure necessary for its further processing, it is first dried and then roasted by gradually raising the temperature. Under the action of heat, the moisture begins to evaporate from the lower layers of the processed material and passes successively through the middle and upper layers, resulting in a process of self-evaporation of oleaginous material (*142*, *144*, *145*). Heating to 50–60 °C causes a sharp decrease in its oil viscosity, while with a further increase in temperature, the viscosity changes are less significant (*142*). At the same time, a temperature range from 40 to 55 °C is the optimal for enzymatic pretreatment of oilseeds, at lower temperatures the process will not be efficient enough, and above 60 °C denaturation of the enzyme protein part and its inactivation will occur (*3,4,6,9,10,13,108-114,117,118,130131,133,134*).

During roasting, the temperature gradually rises from 80 to 105 °C, which causes denaturation of proteins, aggregation of particles, and the material becomes more rigid. Carrying out the process at temperatures above 105 °C is undesirable, as it will intensify the oxidation processes in the obtained oil. The final moisture content of the oil material after roasting has to be 5.0–6.0 % for sunflower seeds, 4.5–5.0 % for flaxseeds and 4.6–6.0 % for cottonseeds (*142*, *144*, *145*).

**Fig. 2:**
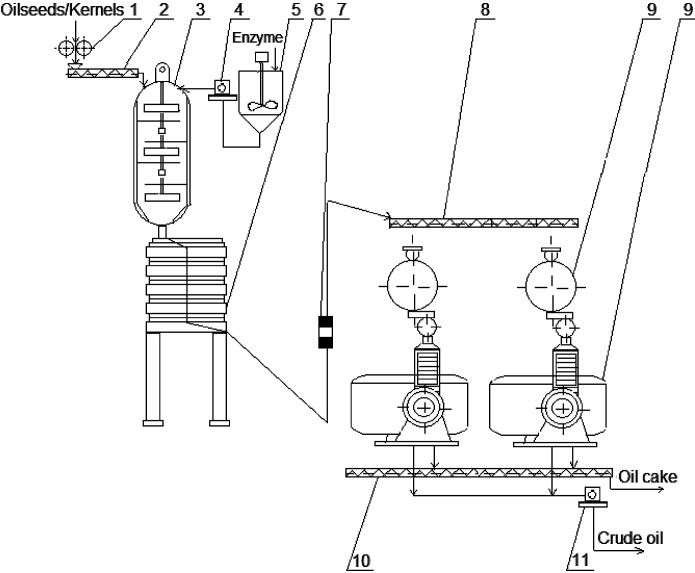
Scheme of process units for enzyme-assisted pressing. Flaking rolls (1), screw steamer (2), mixer expositor (3), pumps (4,11), tank for enzyme solution (5), roaster (6), conveyor belt (7), screw conveyors (8,10), and screw press (9)

A possible application of the enzyme-assisted mechanical pressing in industry is shown in Fig. 2, where the enzymatic treatment of oil-containing material is carried out in the mixer-expositor (3) during cooking, *i.e.* after the stage of seed enzyme inactivation in the screw steamer (2), and before the stage of roasting material in the roaster (6). The enzyme solution with the required pH=4.5–5.5 is prepared in a tank (5) and fed to the mixer-expositor (3). The enzymes added to the oleaginous material are inactivated in the roaster (6).

In the analysis given in the previous section, it was found that 1.5–2.0 h is the optimal duration of the oil-containing material incubation with the enzymes before pressing, excluding time for inactivation and roasting, while the duration of traditional cooking in industry, including inactivation, humidification and roasting, usually is 40–45 min (*108*-*110*, *113*, *114*, *133*, *141*, *142*). A twofold increase of the process duration is a significant disadvantage of this technology and might be difficult to implement in continuous industrial process. However, enzyme-assisted pressing can be effectively used in smaller enterprises with a non-continuous, periodic production.

## CONCLUSIONS

Nowadays, enzymatic pretreatment of oil-containing plant materials and oilseeds is investigated mainly at the laboratory scale and only in a few pilot studies. To transfer this technology to the industry, it has to increase the yield of oil extraction significantly, but not to interfere with the established processes. A key factor is the treatment time, which has a strong influence on technical applicability, commercial feasibility and product quality. Therefore, it is important to optimize the applied enzyme cocktails and the key parameters of the enzymatic pretreatment to keep the process time short, the particle size big enough to maintain porosity for pressing, and the moisture content as low as possible. Other factors such as temperature and pH during enzymatic pretreatment are easier to adapt to increase enzyme performance. The biggest factor is, however, an optimal combination of enzymes for a given substrate that attacks not only the cell wall, but also the membrane oleosome simultaneously and combines different strategies to weaken the cell microstructures for efficient oil extraction. The cell structure is a very complex and multicomponent system and varies with the type of seed. In order to make efficient use of enzymatic pretreatment, its chemical composition has to be investigated and the enzymes have to be selected accordingly. A beneficial outcome of the enzymatic pretreatment might be a reduced pressure during pressing, which reduces the energy demand and costs. The combination of enzymatic pretreatment with mechanical extraction by pressing has the potential to be more eco-friendly than solvent oil extraction methods. Due to the different properties and composition of various oilseeds, each of them needs an individual optimization for pretreatment.

The increased hydrolysis of plant biopolymers can also intensify the recovery of other valuable components, such as carbohydrates, protein isolates, or protein hydrolysates from the plant biomass. In combination with the improved oil recovery, this can ensure waste-free production and promote the efficient use of renewable materials.
